# Network Oscillations Drive Correlated Spiking of ON and OFF Ganglion Cells in the rd1 Mouse Model of Retinal Degeneration

**DOI:** 10.1371/journal.pone.0086253

**Published:** 2014-01-28

**Authors:** David J. Margolis, Andrew J. Gartland, Joshua H. Singer, Peter B. Detwiler

**Affiliations:** 1 Department of Cell Biology and Neuroscience, Rutgers, The State University of New Jersey, New Brunswick, New Jersey, United States of America; 2 Department Physiology and Biophysics and Program in Neurobiology and Behavior, University of Washington, Seattle, Washington, United States of America; 3 Department of Biology, University of Maryland, College Park, Maryland, United States of America; Baylor College of Medicine, United States of America

## Abstract

Following photoreceptor degeneration, ON and OFF retinal ganglion cells (RGCs) in the *rd-1/rd-1* mouse receive rhythmic synaptic input that elicits bursts of action potentials at ∼10 Hz. To characterize the properties of this activity, RGCs were targeted for paired recording and morphological classification as either ON alpha, OFF alpha or non-alpha RGCs using two-photon imaging. Identified cell types exhibited rhythmic spike activity. Cross-correlation of spike trains recorded simultaneously from pairs of RGCs revealed that activity was correlated more strongly between alpha RGCs than between alpha and non-alpha cell pairs. Bursts of action potentials in alpha RGC pairs of the same type, i.e. two ON or two OFF cells, were in phase, while bursts in dissimilar alpha cell types, i.e. an ON and an OFF RGC, were 180 degrees out of phase. This result is consistent with RGC activity being driven by an input that provides correlated excitation to ON cells and inhibition to OFF cells. A2 amacrine cells were investigated as a candidate cellular mechanism and found to display 10 Hz oscillations in membrane voltage and current that persisted in the presence of antagonists of fast synaptic transmission and were eliminated by tetrodotoxin. Results support the conclusion that the rhythmic RGC activity originates in a presynaptic network of electrically coupled cells including A2s via a Na^+^-channel dependent mechanism. Network activity drives out of phase oscillations in ON and OFF cone bipolar cells, entraining similar frequency fluctuations in RGC spike activity over an area of retina that migrates with changes in the spatial locus of the cellular oscillator.

## Introduction

The axons of retinal ganglion cells (RGCs), the output cells of the retina, carry digital messages, encoded as spikes, which tell the brain what the eye sees. The connection between RGCs and the CNS remains functionally intact in retinitis pigmentosa (RP), a group of degenerative retina diseases that attack rod and cone photoreceptors causing blindness in ∼ one in 4,000 people. While RGCs survive the degenerative loss of photoreceptors in RP and retain their intrinsic electrical properties and projection to CNS targets [1]–[7], their spontaneous spike activity switches from a random pattern to a rhythmic one in which bursts of spikes occur at roughly 10 Hz and that persists as the disease progresses from early to late stages [8]–[13]. The possibility of using the retina's output cells to send “visual” signals to the brain and restore vision in patients blinded by retinal degeneration [14], [15] has renewed interest in the properties of RGCs in animal models of RP. To optimize strategies to rescue vision based on this approach it is important to document the properties of pathological RGC spike activity and the mechanisms that give rise to it.

Previous studies have established that spike activity in RGCs in the *rd-1/rd-1* mutant (RD1) mouse, a well studied model of human RP, is driven by rhythmic synaptic input from presynaptic retinal neurons [5], [8], [10], [12] but the extent to which this activity is synchronized is not clear [10], [11], [13].

This issue was examined here by recording from pairs of RGCs in the RD1 retina. In identified alpha RGCs spike discharge was synchronous and in phase when paired recordings where made from cells of the same functional class, i.e. either both ON or both OFF type RGCs. Synchronous oscillations were also present in paired recordings from dissimilar cell types (i.e. ON cell paired with an OFF cell), but bursts of spikes were generated 180 degrees degrees out of phase with respect to each other. This, along with results showing that in RD1 retina A2 amacrine cells generate spontaneous 10 Hz voltage and current oscillations that continue in the presence of synaptic blockers, support the conclusion that the electrically coupled A2 network contributes to the rhythmic synaptic input that drives reciprocal activity in the ON and OFF RGC pathways in retina blinded by degenerative disease.

## Materials and Methods

### Animals

Experimental procedures were similar to previous work [5]. All experiments were conducted in accordance with institutional and national guidelines for animal care using procedures and protocols that were reviewed and approved by the Institutional Animal Care and Use Committee at the University of Washington. All efforts were made to minimize suffering of the mice. Adult C3HeJ mice (*rd-1/rd-1*; RD1; n = 7 for ganglion cell recordings; n = 4 for amacrine cell recordings) were obtained from the Jackson Laboratories (Bar Harbor, ME) and, unless noted otherwise, used at post-natal day (pnd) 40 to 50 (median 44), when their retinas were not responsive to light due to the loss of photoreceptors. Animals were housed in temperature-regulated facilities on a 12/12 hour light/dark cycle and had free access to food and water. As in previous work, mice were not dark adapted for these experiments.

### Tissue preparation and electrophysiological recording: whole mount retina

Mice were killed by cervical dislocation (to avoid potential effects of anesthesia) and eyes removed into room temperature Ames medium (Sigma, St. Louis, MO) equilibrated with 95% O2/5% CO2 (Carbogen), hemisected, and the cornea and lens removed. The resulting eyecup was cut into 2–4 pieces and stored in oxygenated Ames until needed. Retina was isolated by gently teasing it from the pigment epithelium and the vitreous removed before mounting it flat, photoreceptor-side down onto Anodisc filter paper (Whatman, Florham Park, NJ) in a custom recording chamber that was mounted on the stage of a two-photon microscope (see below) and super-fused with warmed Carbogenated (30–34 C) Ames at a rate of 4–8 mL/min.

The flat-mount retina was illuminated with infrared light, imaged using a CCD camera, and visualized on a video monitor. Cells in the ganglion cell layer were targeted for patch clamp recordings based on soma size: alpha cells had large diameter (18–25 µm) somas, and cells with soma diameters <15 µm were classified as non-alpha RGCs. Electrode access to a selected cell was obtained by using an empty patch pipette to micro-dissect the internal limiting membrane above it [16]–[18]. Patch clamp recordings were made using 3–7 MΩ electrodes, and signals were amplified using either Axopatch 200B, Axoclamp 2B or Multiclamp 700b amplifiers. For cell-attached recordings, pipettes contained either Ames solution or standard internal solution (in mM): 120 K-gluconate, 5 NaCl, 5 KCl, 5 Hepes, 1 MgCl_2_, 1 adenosine 5′-triphosphate, and 0.1 guanosine 5′-triphosphate, adjusted to pH 7.4 with KOH, plus 140 µM Oregon Green BAPTA-1 (Invitrogen, Eugene, OR). No differences were found in firing properties measured with either solution. After waiting 2–5 min for the recording to stabilize, spontaneous spike train data were acquired through an ITC-16 interface (Instratech, Port Washington, NY) using software written in Igor Pro (Wavemetrics, Lake Oswego, OR) by Fred Rieke and MATLAB (MathWorks, Natick, MA) by AJG. All chemicals were purchased from Sigma or Tocris (Ellisville, MO).

Following cell attached recording, whole cell access was obtained and RGCs were identified as ON, OFF transient or OFF sustained based on morphology and dendritic stratification depth within the inner plexiform layer (IPL). Dendritic stratification was measured as in a previous study [16], [19]. Briefly, at the end of patch recordings, green fluorescence from Oregon Green BAPTA-1 (OGB-1) filled RGCs was measured using Z-series image stacks and compared to red fluorescence acquired simultaneously from bath-applied Sulforhodamine-101 (SulRh). IPL borders were defined as the first image in SulRh-stained Z-series image stacks to contain no somata. Dendritic stratification was expressed as the location of the peak dendritic fluorescence within the IPL borders.

Fluorescence measurements were made using a custom built two-photon laser-scanning microscope [20] designed around Sutter micromanipulators (Sutter Instruments, Novato, CA) and controlled by CfNT software (written by Michael Muller, MPIMF, Heidelberg). Fluorescence excitation was provided by a pumped infrared laser (Mira; Coherent, Santa Clara, CA) at 905–930 nm, and collected by a 60×1.0 NA water immersion objective (Nikon, Tokyo, Japan). Custom band-pass filters (Chroma Technology, Rockingham, VT) directed green (535±50 nm) and red (622±36 nm) fluorescence emission to two independent photomultiplier tubes (Hamamatsu, Hamamatsu City, Japan). The green channel was used for Ca^2+^ indicator fluorescence, and the red channel was used to monitor fluorescence from bath-applied SulRh.

### Tissue preparation and electrophysiological recording: retinal slices

Mice were anesthetized with isofluorane, decapitated, and enucleated. Retinae were isolated into bicarbonate buffered Ames' medium (Sigma) equilibrated with Carbogen. Retinae then were embedded in low-melting temperature agarose (Sigma type VIIA, 3% in a HEPES buffered saline), and slices (200 μm) were cut on a vibrating microtome (Microm Corp.). Slices were stored in Carbogen-bubbled Ames' medium until use. Retinal slices were super-fused with Ames' medium (∼32–34°C) to which drugs were added as noted. Picrotoxin (100 μM), strychnine (0.5 μM), Dinitroquinoxaline-2,3-dione (DNQX, 25 μM) and APV (50 μM) were added to block GABA-A receptor-, glycine receptor-, AMPA/kainate receptor-, and NMDA receptor-mediated currents, respectively. Tetrodotoxin (TTX, 0.5 μM) was added to block voltage-gated Na^+^ channel-mediated currents. Pipettes for whole cell current and/or voltage clamp recording were filled with the following solution (in mM): 95 KGluconate, 15 KCl, 5 NaCl, 10 HEPES, 0.2 EGTA, 8 Tris-Phosphocreatine, 4 MgATP, and 0.4 NaGTP. Access resistances were typically <25 ΜΩ and were not compensated. Recordings and data analysis were performed using a MultiClamp 700A amplifier, an ITC-18 A/D interface, and IgorPro software as described previously (e.g., Tian et al. 2010).

### Data analysis

Electrophysiological data were analyzed in Igor Pro and MATLAB. Power spectra were computed from long (>15 s) current or voltage sweeps using built-in routines in Igor that measured power in 1.22 Hz bins. Optical data were analyzed in Igor Pro and ImageJ (http://rsb.info.nih.gov/ij/).

### Spike train analysis

#### Spike time rasters

Extracellularly recorded spike trains were converted to rasters of spike times defined as a threshold crossing of 5 times the standard deviation of the first derivative of the extracellular voltage, and verified by eye.

#### Correlograms

Interval correlograms were created using the spike times of two spike trains (cell 1, cell 2) with n_cell1_ and n_cell2_ being the total number of spikes in the trains recorded from each of the two cells. Intervals were calculated by subtracting the spike times of each spike in cell 2 from every spike in cell 1 yielding a total of n_cell1_ x n_cell2_ intervals and a histogram constructed from the intervals using 2 ms wide bins from −500 ms to +500 ms. A negative interval signifies a spike in cell 2 that occurs after a spike in cell 1. The histogram contains the number of intervals that fall in each bin. To compare correlograms across different cells or pairs of cell counts per bin were normalized by dividing by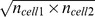.

An auto-correlogram is a correlogram in which the first and second spike trains are the original and a duplicate, respectively, of the cell's spike train. In this case the bin containing intervals equal to zero has exactly n_cell1_ counts. Subsequent normalization gives this “zero lag” bin a value of 1.

#### Shuffling

A spike train generated by a random Poisson process in which the probability of spiking remains constant over time, will have a flat auto-correlogram save two qualifications: the “zero lag” time bin will have a normalized value equal to 1 and there are proportionately fewer long intervals since a spike train of finite length has fewer long intervals and hence can have no intervals longer than twice the length of the spike train. Any significant deviation from this auto-correlogram of a random spike train indicates variation in the spike probability over time. Variation could include a rhythmic spike probability, which would result in rhythmic spike bursts as observed in RD1 alpha RGCs. To determine which deviations are statistically significant we used a permutation test in which the spike train is split into 1 sec chunks that can be shuffled prior to creating the auto-correlogram. By choosing a chunk width of 1 sec, the statistics of the spike train and those of the ∼10 hz oscillation are preserved, while synchrony on a long timescale is destroyed. The auto-correlogram is then created using the original spike train and a chunked and shuffled duplicate. The resulting auto-correlogram will still have a small peak at zero, since by-chance one of the chunks may still be aligned with that of the original train. However, for the most part, the correlogram will be flat, like the auto-correlogram of a random spike train. The statistical test then involves repeating the shuffled auto-correlogram several hundred times. Any bins of the original histogram with values that are greater than or less than 95% of the values in the shuffled cases are considered to be statistically significant. This test asks for each bin whether it is likely, given a rhythmic spike train, that you would get as big (or small) a value by chance alone.

#### Sliding windows

A sliding window was used to assess how aspects of the correlograms change over time. The analysis consists of creating a correlogram for a 5 sec “window” or subset of a longer recording, calculating useful parameters from the correlogram (i.e. correlation strength, characteristic frequency, phase) and then repeating the process, each time sliding the window by 0.1 to 0.5 sec. A window width of 5 sec was used because it contains close to 50 spike bursts of a RGC oscillating at 10 Hz, which is enough data in order to accurately estimate frequency and phase. The increment of 0.5 sec means that windows have an 80% overlap or about 40 spike bursts in common. This method of oversampling ensures that no changes in the parameters will be missed. Note that to estimate stability in any of the parameters by using their standard deviation should only be computed using contiguous, non-overlapping windows.

#### Peak finding

All of the parameters of a correlogram require identifying the amplitude and locations of peaks. Peaks were estimated from smoothed versions of the correlograms using a 10 point sliding average in both directions (to avoid a time shift). Finding the maximum value over a specified window identified peaks. The correlation strength was determined as the peak to trough amplitude of the correlogram.

## Results

### Ganglion Cell type identification and targeting

**Figure 1:**
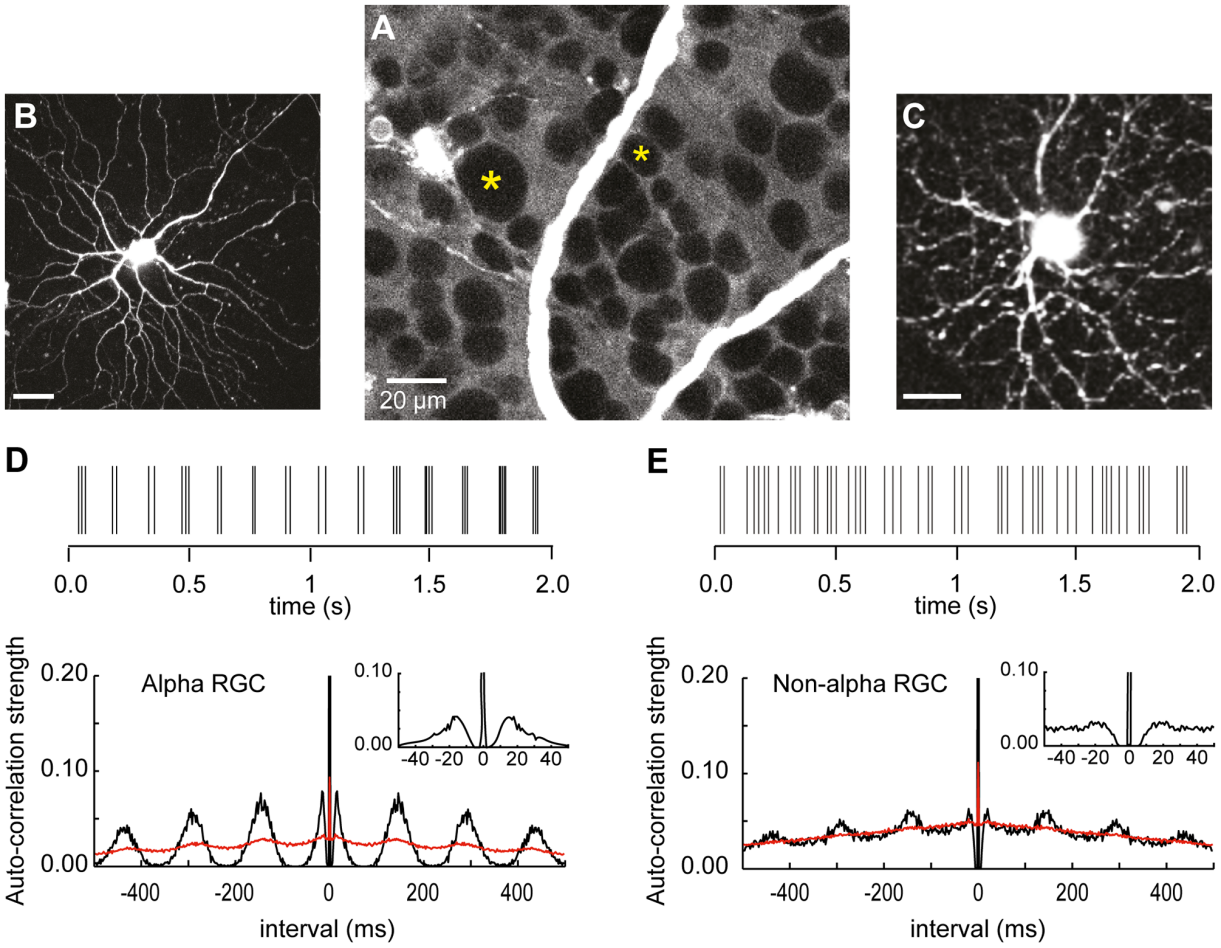
Cell targeting and spike train auto-correlograms. **A.** Fluorescence images of retina in presence of bath applied sulforhodamine, a counterstain showing intact cell bodies (dark) on lighter fluorescent background with examples of a large (alpha) and small (non-alpha) somas RGCs indicated by asterisks. Two-photon Z-projection images of alpha (**B**) and non-alpha (**C**) RGCs following internal whole cell dialysis with pipette filling solution that contains 140 uM OGB-1. Scale bars 30 and 15 um, respectively. Spike train rasters recorded from alpha (**D**) and non-alpha (**E**) RGCs and corresponding auto-correlograms (black traces) based on 166 s records, 2 ms bin widths. Red traces are auto-correlograms using shuffled spike trains (see Methods & Materials). Insets show auto-correlograms on expanded time scales to illustrate reduced spike probability during a ∼5 to 10 ms refractory period following a spike.

The results are based on cell-attached extracellular recordings of spontaneous spike trains from 44 retinal ganglion cells (RGCs) in whole-mount retina from RD1 mice blinded by photoreceptor degeneration. RGCs were targeted for recording based on soma size as assessed using 2-photon laser scanning fluorescence imaging with SulRh as an extracellular florescent counter stain [21]. Under these conditions cell bodies appear as black objects outlined by a background of SulRh fluorescence (Fig. 1A). The alpha class of RGCs comprises cells with the largest soma diameters (18–25 µm) [22]; cells with soma diameters ≤15 µm were designated as non-alpha RGCs with less extensive dendrites than those of alpha-type RGCs Following a period of data collection by continuous (2 to 5 min) cell-attached extracellular recording of spontaneous spike activity a second electrode was advanced to establish a whole cell recording and to fill the targeted cell with internal solution containing a fluorescent dye (OGB-1) for morphological characterization using full field 2-photon fluorescence imaging (Fig. 1B,C).

In wild type retina alpha RGCs are either excited by light onset (ON cells) or light offset (OFF cells). The two cell types can also be identified on the basis of the depth that their mono-planar dendrites stratify in the IPL of the retina, with the dendrites of ON and OFF alpha RGC terminating in the inner and outer sublamina of the IPL, respectively [16], [23]. The differences in stratification depths are retained in RD1 retina [6], [24] and provide a way of classifying alpha RGCs as ON or OFF cell types without the benefit of a light response [5]. The OFF alpha cells can be further divided into two subcategories that may be distinguished in functionally intact WT retina by whether they generate a transient (OFF-T) or sustained (OFF-S) burst of action potentials at light offset and in blind RD1 retina by whether their dendrites stratify in the inner or outer portion of the OFF sublamina of the IPL, respectively.

On the basis of soma size our set of 44 RD1 RGCs included 29 alpha cells and 15 non-alpha cells. Alpha RGCs were further segregated on the basis of different dendritic arbor depths into 7 ON, 17 OFF and, as a consequence of unsuccessful dye incorporation and fluorescence imaging, 5 alpha RGCs that were of unknown subtype. Nine of the 17 OFF alpha cells were sub-classified using stratification depth as OFF-T (n = 4) and OFF-S (n = 5) alpha RGCs.

Recordings from non-alpha cells were often paired sequentially with a maintained recording from a single alpha cell (see “Paired recording versus distance analysis”, below). As a consequence only a small number of non-alpha RGCs were successfully filled with dye by whole cell dialysis and imaged. Of the four that were two had mono-stratified dendrites (one each in the ON and OFF sublamina) and two were bi-stratified.

### Rhythmic spike activity (auto-correlation)

#### Alpha ganglion cells

In the RD1 retina alpha cells generated spikes spontaneously (Fig. 1D) at average rates that in different alpha cells ranged from 9 to 39 (mean 22, n = 29) Hz (Table 1). In all cases spontaneous spike activity included rhythmic bursts of spikes (Fig. 1D) that contained 2–10 spikes per burst. These features of alpha cell spontaneous activity were apparent in their auto-correlograms, in which a copy of the recorded spike train is made and all the time intervals between every spike in the recorded train and every spike in the duplicate train are tabulated and plotted as a histogram of counts per inter-spike time interval (lag time) over a window from 0 to +/−500 ms (Fig. 1D). Since every spike in the recorded train is perfectly correlated with the occurrence of a spike at exactly the same time in the duplicate train, normalizing the counts per histogram bin by dividing by the total number of spikes in the recorded train (see Methods) gives a measure of auto-correlation strength that extends from perfect correlation (1.0) at zero lag time to no correlation (0).

**Table 1:** Properties of RGC spiking activity in RD1 retina. ON-, OFFT- and OFFS-Alpha refer to types of alpha RGC as described in the main text. OFFUnk-Alpha refers to OFF-Alphas that could not be typed as OFFT or OFFS, and Unclassified cells were not classified as Alpha or Non- Alpha.

Parameter (Mean ± SEM)	ON-Alpha (n = 7)	OFFT-Alpha (n = 4)	OFFS-Alpha (n = 5)	OFFUkn-Alpha (n = 8)	Unclassified (n = 5)	Non-Alpha (n = 15)
Spike Freq. (Hz)	21.5±3.2	22.4±1.9	28.4±3.0	18.1±2.7	24.7±3.2	6.8±1.7
Characteristi Freq. (Hz)	8.3±0.7	8.7±1.2	9.0±0.8	7.7±0.7	9.5±0.6	7.9±0.5
Autocorr. Strength	0.16±0.02	0.15±0.02	0.13±0.02	0.11±0.01	0.16±0.05	0.01±0.05

The rhythmicity of spike bursts is seen as periodic variation in auto-correlation strength with a characteristic frequency (CF): taken as 1/the time interval between sequential peaks in the auto-correlogram. In 29 different alpha cells CF ranged from 5 to 11 Hz, with an over all mean of 8.7 Hz that was the same in both ON and OFF alpha cells, with no distinction between OFF cells classified as sustained or transient subtypes (Table 1).

The variation in correlation strength was largest in the vicinity (±50 ms) of zero lag time and fell off as the inter-spike time interval increased (Fig. 1D). This could be the consequence of there being relatively fewer long spike intervals over a finite record duration or variability in the rhythmicity that would add to the jitter in burst timing. In different alpha RGCs the amplitude of the change in auto-correlation strength, measured from trough to peak, ranged from 0.11 to 0.16 with no significant difference between ON and OFF alpha cell types (Table 1).

To assess the statistical significance of the rhythmicity each recorded spike train was broken into second long segments that were shuffled (see Methods) in the duplicate train before creating the auto-correlogram. Shuffling in this way preserved the fine structure in the bursting activity but removed the continuous periodicity (red trace Fig. 1D). Variations in correlation strength above or below the red shuffle line are therefore considered significant changes in spike probability rather than the consequence of a random process.

The presence of spontaneous bursts of spike activity in the alpha cell spike train is also apparent in the auto-correlogram as an increase in correlation strength at short interval times (±15 to 40 ms) that correspond to the distribution of time intervals between the spikes in a burst (Fig. 1D). Closer examination of the auto-correlogram in this time window (Fig. 1D insert) shows that in this example there are no spikes generated at intervals less than ∼5 ms, indicating that spikes in RD1 alpha RGCs are followed by a refractory period during which there is a decrease in spike probability.

#### Non-alpha ganglion cells

In the RD1 retina, ganglion cells classified as non-alpha cells were, like their alpha cells counterparts, spontaneously active (Fig. 1E), albeit at a lower mean rate (Table 1). They generated spike trains with rhythmic variations in auto-correlation (Fig. 1E) that were smaller in amplitude than in alpha RGC but occurred at the same characteristic frequency (Table 1). Rhythmic spike activity in non-alpha cells commonly consisted of only one or two spikes and was less likely to include bursts of spikes than alpha cells. This can be appreciated by comparing the auto-correlograms of non-alpha and alpha cells in the vicinity of zero lag time; the prominent lobes on either side of t = 0 that represent the distribution of interval times between the spikes in a burst as seen in the alpha cell are absent in the non-alpha RGC (Fig. 1D,E inserts).

### Paired recordings (cross-correlation)

The presence of periodic variation in auto-correlation strength in RD1 alpha and non-alpha RGCs spike trains is consistent with them being driven rhythmically by synaptic input from pre-synaptic elements, as shown in previous studies [3], [5], [8], [10]–[13] in which the synaptic input currents as well as the rhythmic spike discharge they give rise to were eliminated by treatment with a cocktail of glutamatergic and GABA/Glycine antagonists. To learn more about the properties of the rhythmic variations in spike activity, cross-correlation was used to examine the relationship between spontaneous spike trains in pairs of simultaneously recorded RGCs. Paired recordings often were carried out by maintaining the recording from one of the alpha cells in a pair with a stationary electrode while using the second electrode as a rover to record periods of spontaneous spike activity from a variety of RGCs at different distances from the fixed recording site (see “Paired recording versus distance” analysis, below). Simultaneous recordings were not made from pairs of non-alpha RGCs.

While the characteristic frequency of the rhythmic variation in autocorrelation strength ranged from 5 to 11 Hz across cells [5], [10], the characteristic frequency of the auto-correlation of spike trains recorded simultaneous from pairs of RGCs were virtually identical for both alpha/alpha pairs and alpha/non-alpha pairs (see “Characteristic frequency of alpha and non-alpha RGCs and cross-correlogram parameters” analysis, below).

**Figure 2:**
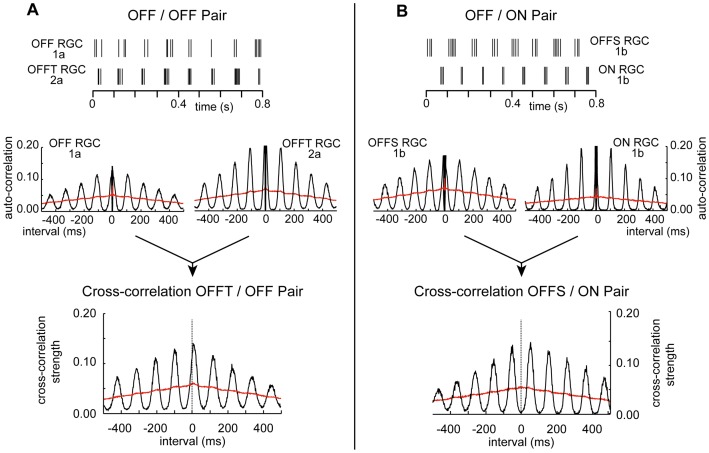
Auto- and cross-correlograms of spike trains recorded from OFF/OFF (A) and OFF/ON (B) alpha RGCs pairs. Rasterized spike trains recorded simultaneously from a pair of OFF alpha RGCs (**A**) and from an OFF and ON alpha RGC pair (**B**), showing in and out of phase spike generation, respectively. Row of four graphs below raster traces show auto-correlograms of spike trains recorded from the two cells in **A (left**) and **B (right)**, total record lengths of 181 and 121 s, respectively. Bottom plots are cross-correlograms for the OFF/OFF (**left**) and OFF/ON (**right**) alpha RGC pairs. In all correlograms black and red traces plot auto- and cross-correlations of original and shuffled spike times, respectively. OFF alpha cells identified as sustained or transient subtypes labeled OFFS and OFFT, respectively.

**Figure 3:**
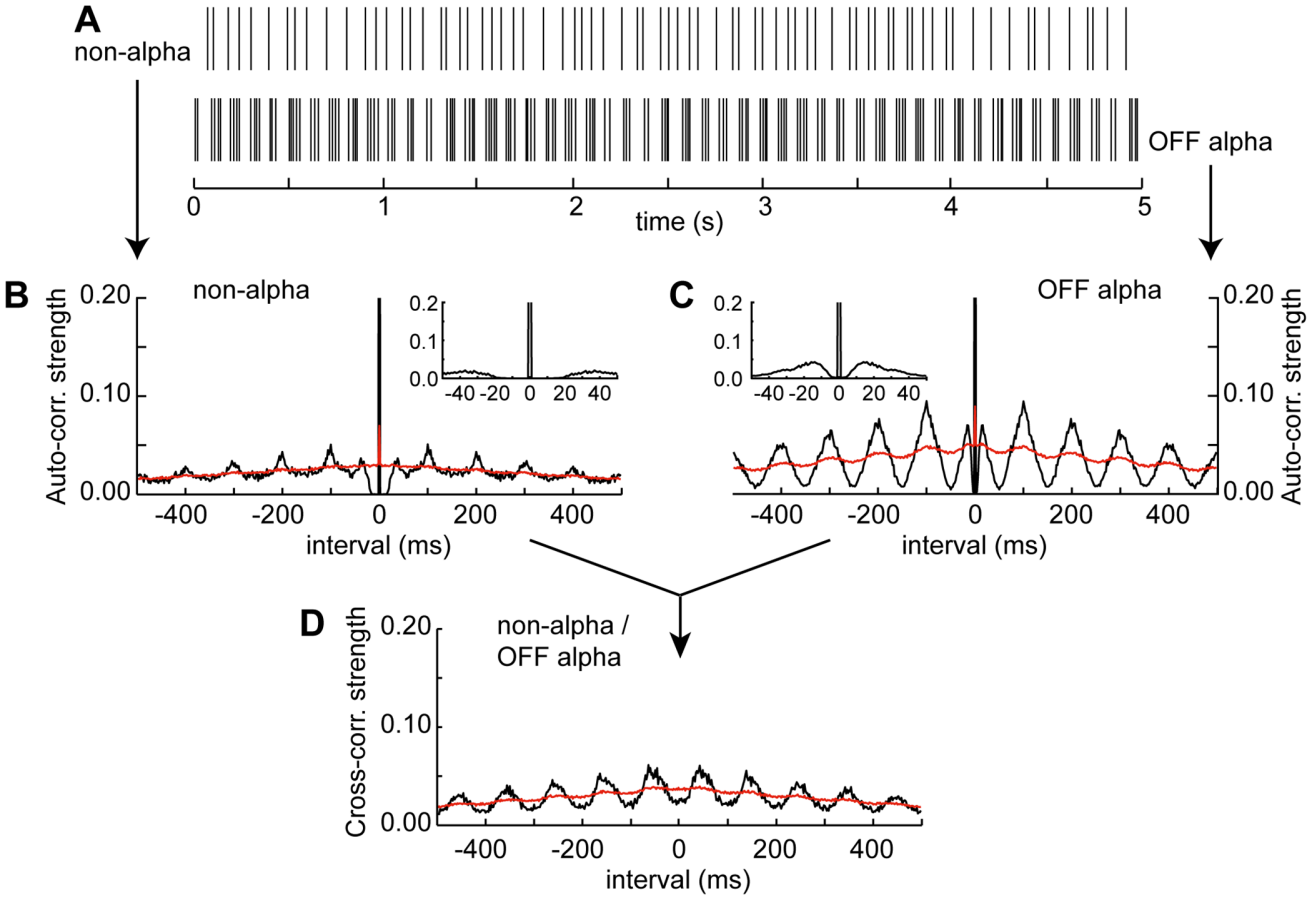
Correlation analysis of spike trains recorded from an alpha and non-alpha RGCs. **A.** Rasterized spike trains recorded simultaneously from a non-alpha (top trace) and OFF alpha RGC. **B, C.** Auto-correlograms of spontaneous spike activity in non-alpha and OFF alpha RGCs in A, respectively. Inserts show auto-correlation on expanded time scale. **C.** Cross-correlogram of spike trains recorded from RGCs in part A, total record lengths 287 s. In all panels black and red traces plot auto- and cross-correlations of original and shuffled spike times, respectively.

The cross-correlogram was calculated in the same way as described above for the auto-correlogram except that the two spike trains were recorded simultaneously from different cells rather than from a single cell where the recorded spike train and its duplicate served as the two trains. The histogram was scaled by dividing the counts per bin by the square root of the product of the total number of spikes in the two trains (see Methods) to give a measure of cross-correlation strength on a scale that is equivalent to the scale used for auto-correlation strength. The cross-correlogram of pairs of alpha RGCs as well as pairs of alpha and non-alpha cells showed periodic variation in correlation strength that declined in amplitude with increasing lag time (Figs. 2, 3). The oscillations in cross-correlation strength were at the same frequency as the average characteristic frequency (CF_avg_) of the periodic variations in the auto-correlograms of the two cells (true for alpha-alpha and alpha-non-alpha pairs; see also “Characteristic frequency of alpha and non-alpha RGCs and cross-correlogram parameters” analysis, below). The maximum amplitude of the variation in cross-correlation strength was about twice as large in alpha-alpha pairs than in alpha-non-alpha pairs (Figs. 2, 3), indicating more robust spike synchrony in alpha than non-alpha RGCs.

**Figure 4:**
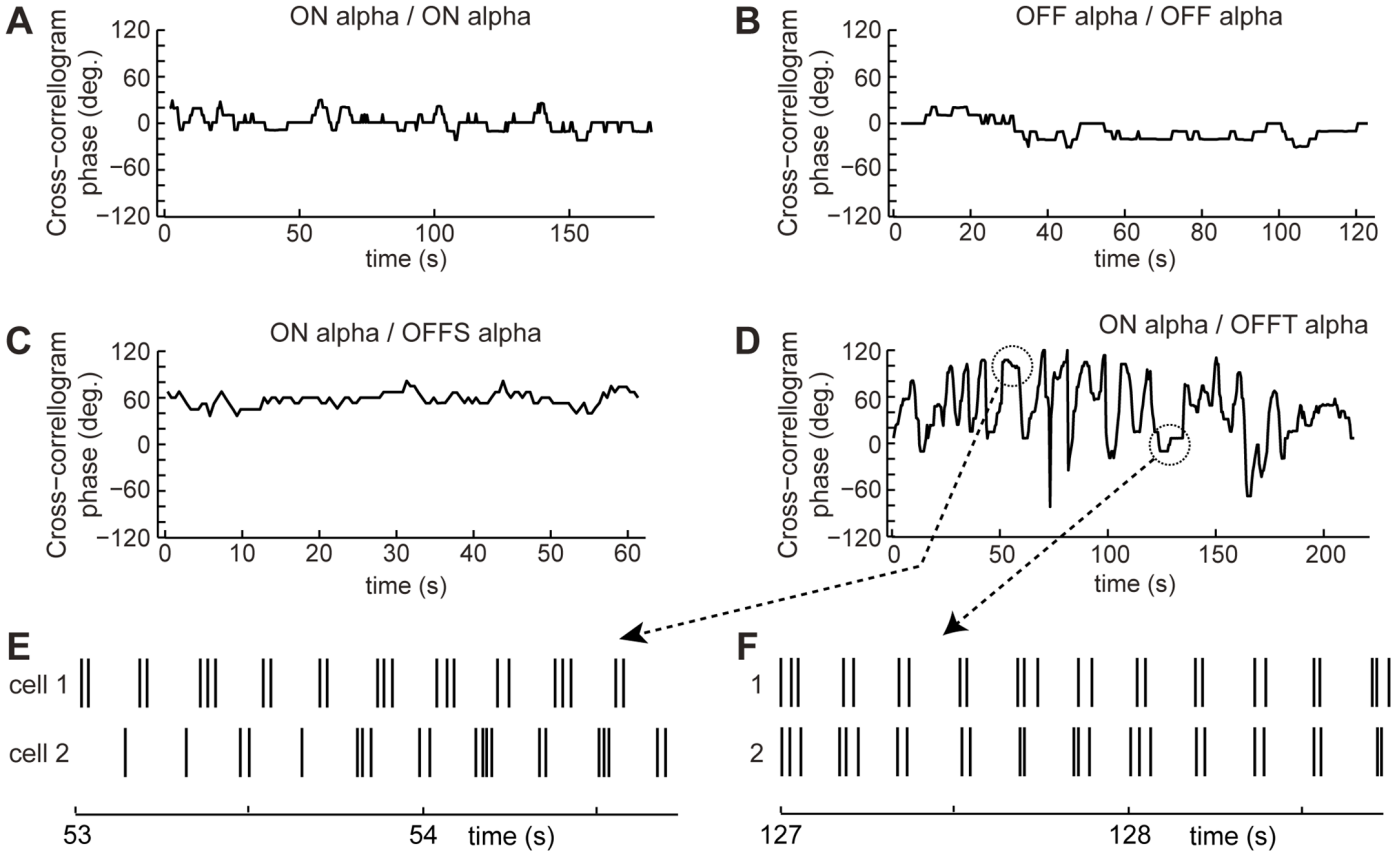
Phase stability in spike firing in paired recordings. The phase relationship of spike generation in trains recorded simultaneously from pairs of like-type alpha RGCs, i.e. both ON cells (**A**) and both OFF cells (**B**) and from un-like type alpha RGCs, i.e. an ON and OFF cells with examples showing stable and un-stable phase relationships, **B, C**, respectively. Phase was calculated from cross-correlograms using a 5 s sliding window that was incremented by 0.5 s. Spike trains during periods of reversed phase are shown by traces in **E, F**. OFF alpha cells identified as sustained or transient subtypes labeled OFFS and OFFT, respectively.

**Figure 5:**
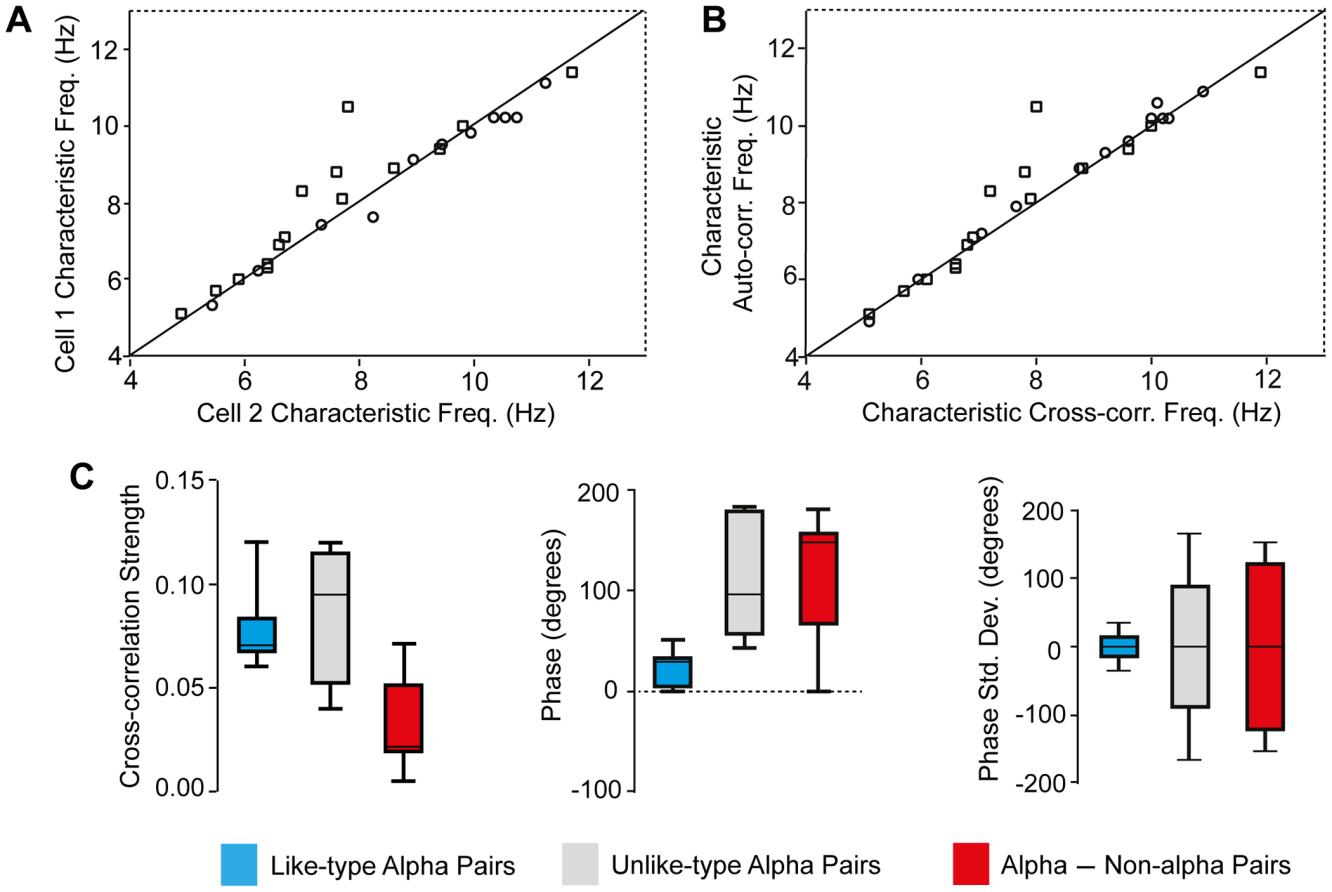
Characteristic frequency of alpha and non-alpha RGCs and cross-correlogram parameters. **A.** Characteristic frequency (inverse of the time interval between sequential peaks in the auto-correlogram) of rhythmic activity based on auto-correlation of spike trains recorded simultaneously from two cells, either a pair of alpha RGCs (open circles; n = 12) or a alpha and non-alpha RGC (closed squares). Solid line represents perfect correspondence between ordinate and abscissa. **B.** Characteristic frequencies of cell pair spike trains based on autocorrelation versus cross-correlation for same cell pairs as in A. **C.** Column graphs of cross-correlation strength (peak-to-trough amplitude), phase and phase standard deviation for like and un-like cell type, i.e. ON vs OFF, alpha pairs (n = 8 and 4, respectively) and alpha-non-alpha pairs (n = 15), blue, gray red columns, respectively. Column whiskers indicate minimum and maximum values, bottom and top of filled section marks intra-quartile range with boundaries at 1/4 of values equal to or less than 25th quartile and 3/4 values equal to or less than 75th quartile, respectively. Horizontal line is the mean of all values in each category.

It was clear from visual inspection of the spike rasters that the rhythmic bursts of spikes in paired recordings were in phase in like types of alpha cells (OFF/OFF) and out of phase in pairs of dissimilar types (ON/OFF) of alpha cells (Fig. 2). The location of the peak of the cross-correlogram on the time axis (L_peak_) provides a measure of the phase relationship (ψ) between the rhythmic spike discharge in the two cells as given by:
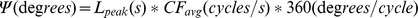



To evaluate the stability of the phase relationship between spike trains over the duration of the period of paired recordings ψ was calculated continuously for a 5 second sliding (0.1 s steps) window (see Methods & Materials) for like- and un-like types of alpha RGC pairs (Fig. 4) and for pairs of alpha and non-alpha cell types (Fig. 5). The variations in the sliding window phase, as measured by its standard deviation, were much smaller in pairs of like-type alpha RGCs than in ON/OFF alpha pairs or alpha/non-alpha pairs. The spike trains were stably out of phase (mean phase 178°) in two of the four ON-OFF pairs (Fig. 4C) and unstable in the other two pairs in which spike discharge in the two cells fluctuated between being in and out of phase with respect to each other (Fig. 4D–F).

The phase relationship between spike trains in alpha and non-alpha RGCs was typically unstable with persistent variations in phase (Fig. 5C) but fluctuations between periods when spikes were in versus out of phase were harder to recognize because of the lower rate of spontaneous spike production in non-alpha RGCs and the inherently weaker variations in cross-correlation strength. The phase shift between the spike trains of pairs of like type alpha cells (i.e. ON/ON or OFF/OFF cells) was significantly smaller (p<0.001) than the phase shift between trains in dissimilar types of alpha cells or between alpha and non-alpha RGCs (Fig. 5C). We have no evidence that there was a difference in the correlation strength of ON-ON and OFF-OFF pairs.

### Paired recording separation distance

**Figure 6:**
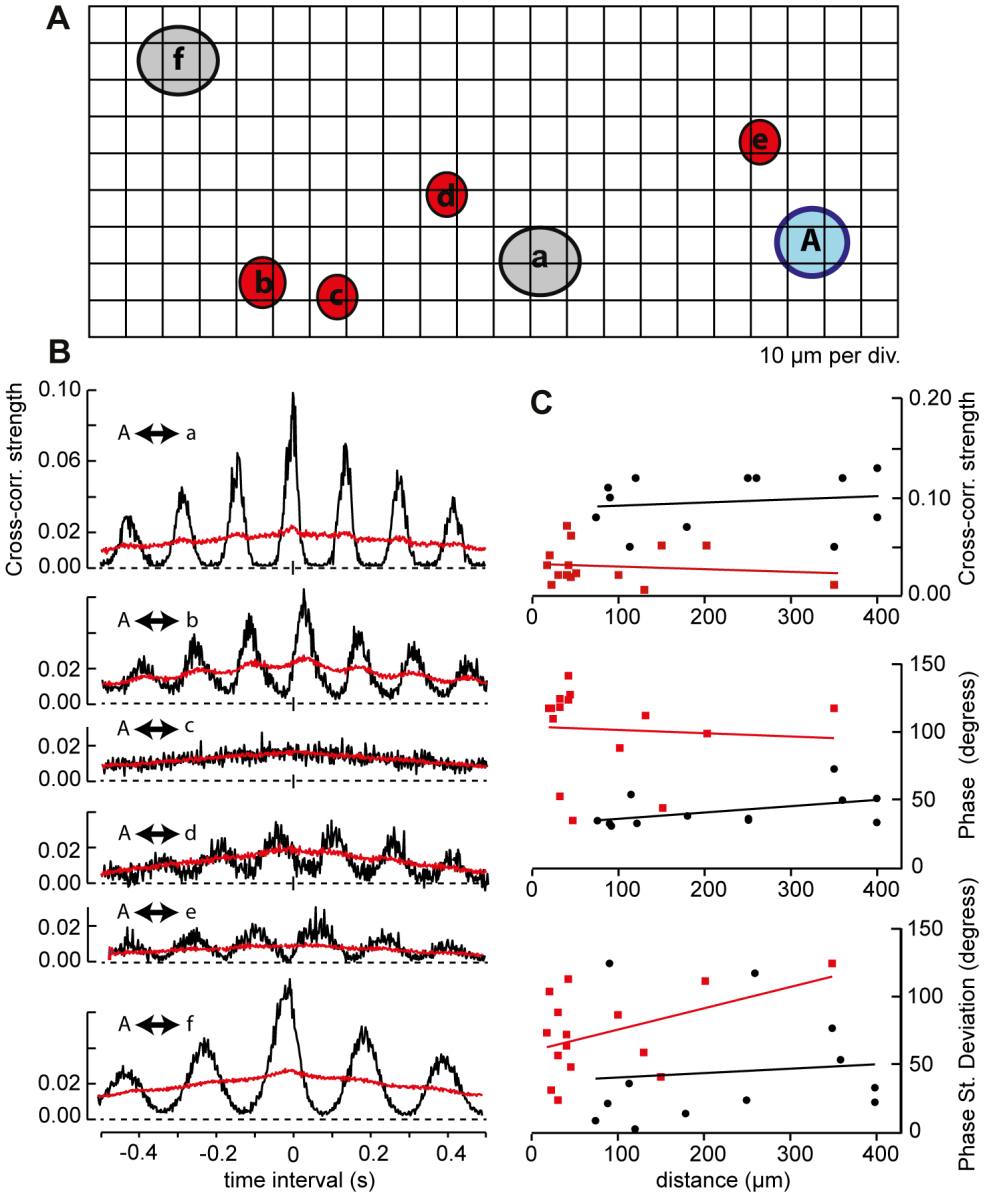
Paired recording versus distance. **A.** Schematic showing spatial relationship of recordings made with a stationary electrode in alpha RGC A (blue) paired with recordings made sequentially from 6 other cells (alpha cells gray, non-alpha cell red) in order of (**a**) to (**f**). **B**. Cross-correlograms of spike trains recorded from each of the six pairs. Record lengths (s) were (**A** to **F**):120, 151, 181, 121, 211, 301. In all panels black and red traces plot cross-correlations of original and shuffled spike times, respectively. The scale bar in the top panel of part B indicates the p = 0.05 level of significance for the difference between the original and shuffled traces, differences larger than the scale bar have proportionately greater levels of significance (p<0.05). **C.** Relationship between separation distance and cross-correlation strength, phase and phase standard deviation for all alpha/alpha (filled black circles) and alpha/non-alpha RGCs pairs (filled red squares). The slopes of linear regression lines fitted to each data set were not significantly different than zero with p values ranging from 0.13 to 0.86.

The separation between pairs of recorded cells ranged from 74 to 400 µm (mean 223 +/−39 µm, n = 12) for alpha/alpha pairs and from 17 to 350 µm (mean 83+/−24 µm, n = 15) alpha/non-alpha pairs. None of the cross-correlation parameters, i.e. correlation strength, phase and phase stability) were dependent on the distance between recorded pairs of either alpha-alpha or alpha-non-alpha cells (Fig. 6). It is important to note that these recordings were made sequentially (with cell A held and common to all pairs); variability in the characteristic frequency of oscillatory activity could reflect natural variability over time.

### Oscillatory activity in A2 amacrine cells

The results presented above are consistent with the rhythmic discharge in RD1 RGCs being driven by oscillations in synaptic input rather than by the intrinsic properties of the cells [3], [5], [8], [10]–[13]. To investigate the source of the synaptic drive whole cell recordings were made from A2 amacrine cells in RD1 retina slices. The A2 amacrine cell was considered an important candidate to evaluate since the coupled network of A2s is capable of coordinating activity between in ON and OFF pathways on a distance scale consistent with the range of separations between members of the recorded RGC pairs [25]. In addition the A2 mediated excitation of ON cone bipolars by electrical coupling and simultaneous glycinergic inhibition of OFF cone bipolar cells could account for spike discharge being in phase between pairs of like-type alpha RGCs (ON/ON and OFF/OFF pairs) and out of phase for un-like (ON/OFF) cell types (as in Fig. 2).

**Figure 7:**
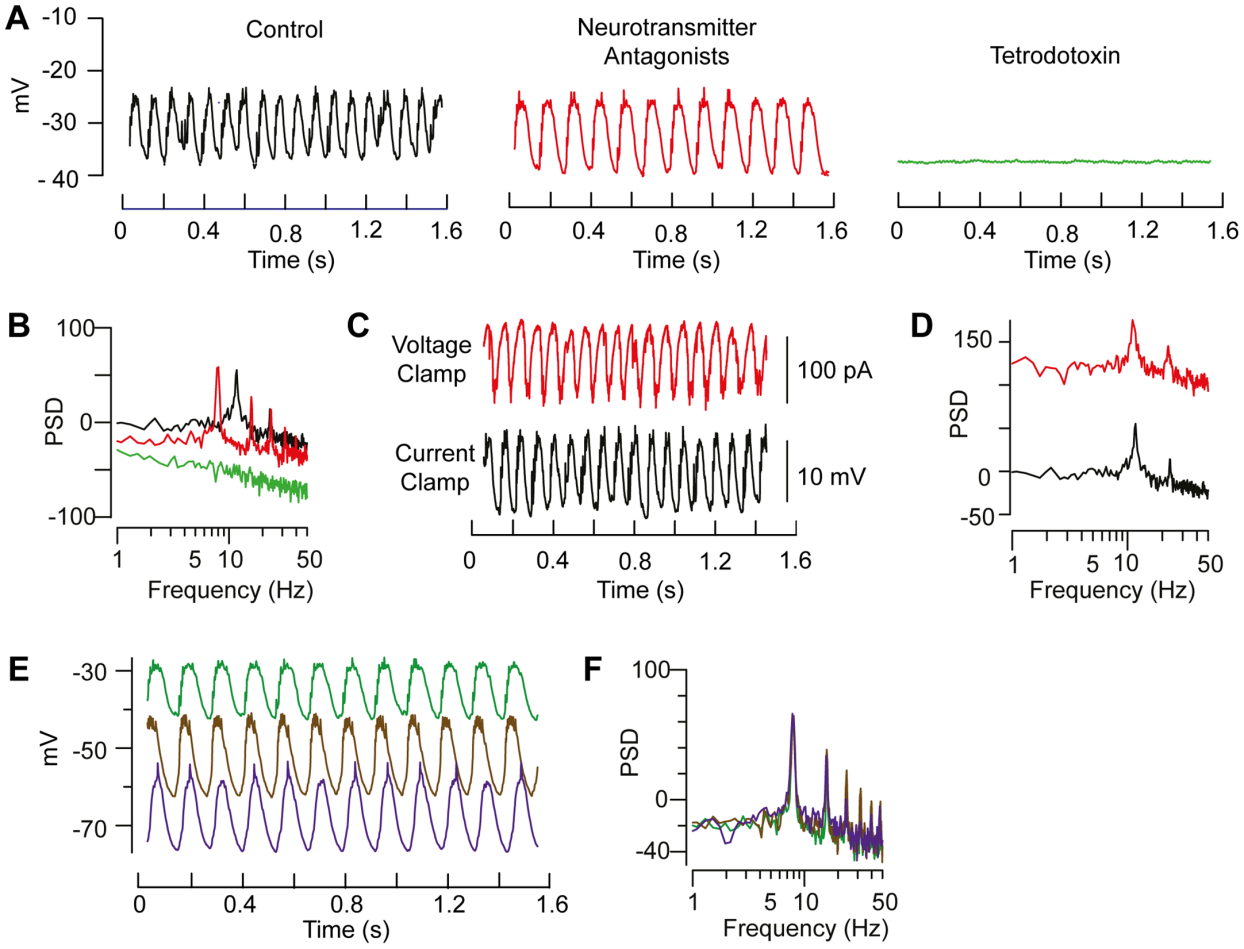
A2 amacrine cell recordings from RD1 retina slices. **A.** Current clamp recording of spontaneous membrane potential (V_m_) oscillations in Ames' solution (control, black trace), with addition of a cocktail of APV, DNQX, picrotoxin, strychnine (neurotransmitter antagonists, red trace), followed by added tetrodotoxin (green trace); mean V_m_  = −34 mV. **B.** Power spectral density (PSD) of membrane potential oscillations under the same three conditions and color code. **C.** Oscillations persist in presence of neurotransmitter antagonists under current- (black) and voltage-clamp (red, V_hold_  = −80 mV) conditions. **D.** Power spectra of oscillations recorded in current- (black) and voltage-clamp (red). In presence on neurotransmitter antagonist membrane potential had little effect on the amplitude of voltage oscillations (**E**) and no affect on their frequency (**F**).

Like RGCs, A2s in the RD1 retina showed large-amplitude (∼10 mV) spontaneous oscillations in membrane potential at a frequency ∼10 Hz (10.6±0.7 Hz; n = 7; Fig. 7A,B). Voltage oscillation frequency in A2s closely matched the frequency of burst firing in alpha RGCs (Table 1).

### A2 activity reflects an intrinsic (network) oscillation

What generates oscillatory activity in A2s? In WT retina, A2s are part of an electrically coupled network of other A2s and ON cone bipolar cell synaptic terminals. A2s also receive glutamatergic input from rod bipolar cells as well as inhibitory input from other amacrine cells. Two lines of experimental evidence, however, indicate that the oscillations originate from the coupled A2s themselves. Firstly, oscillations in membrane current were recorded in A2s in the voltage-clamp configuration (holding potential  = −80 mV), and these had the same frequency as membrane voltage oscillations recorded in the same cells (Fig. 7 C,D; 10.7±0.6 Hz vs 10.7±0.5 Hz; current vs. voltage; 4 of 4 A2s examined). Secondly, oscillations persisted in the presence of a combination of excitatory and inhibitory synaptic antagonists (see Methods) with a decrease (∼28%) in frequency but little change in amplitude (Fig. 7A,B). The decrease in frequency could reflect changes in the excitability of A2s and/or A2-ON cone bipolar networks and remains an area for future research.

The oscillations in A2 voltage were abolished by application of 0.5 µM TTX (Fig. 7 A,B), which blocks voltage-gated Na^+^ channels and Na^+^ channel-dependent bursting in WT A2s [26], [27]. This combined with the observations that oscillations do not require synaptic input (Fig. 7A,B), that they are observed in A2s under voltage-clamp (Fig. 7C,D), and that their frequency is independent of the membrane potential of the recorded A2 (Fig. 7E,F) support the conclusion that the oscillations are generated at the network level by a Na^+^ channel-dependent mechanism.

## Discussion

The autocorrelograms of spontaneous spike trains show that there is low frequency (∼10 Hz) rhythmic spike discharge in alpha and non-alpha RGCs in the RD1 retina in agreement with several previous studies [3], [5], [8], [10]–[13]. The rhythmic discharge of RGC spikes is driven by 10 Hz oscillations in synaptic input from both excitatory and inhibitory presynaptic neurons, i.e. glutamatergic bipolar cells and GABA/glycinergic amacrine cells [5], [8]. Bursts of spike discharge occurred synchronously in simultaneous recordings from pairs of RD1 RGCs. The cross-correlation of spontaneous spike discharge was stronger in trains of spikes recorded from pairs of alpha RGCs than from pairs consisting of an alpha and non-alpha RGC (Figs. 2,3,5C). The presence of synchronous firing in both cell types (alpha and non-alpha) indicates that both get common input from a presynaptic generator that controls all the RGCs in a patch of RD1 retina on the scale of a few hundred µm, in agreement with the size of the extracellular local field potentials (LFPs) reported by Menzler and Zeck [10]. The phase delay between synchronous discharge in cell pairs was less (closer to zero) in pairs of like-type alpha cells (ON/ON and OFF/OFF RGC pairs) than in dissimilar pairs of either ON/OFF alpha RGC s or alpha/non-alpha pairs (Fig. 5C).

To account for the correlated firing in ON/ON and OFF/OFF pairs of alpha RGCs the common presynaptic oscillator must drive synchronous activity in ON and OFF bipolar cells and as well as in inhibitory amacrine cells in order to produce the observed oscillations in inhibitory synaptic input to RD1 RGCs [5], [8]. The synchronous but out of phase coordination of spike discharge in ON/OFF alpha cell pairs suggests that the presynaptic oscillator excites one pathway when it inhibits the other.

**Figure 8:**
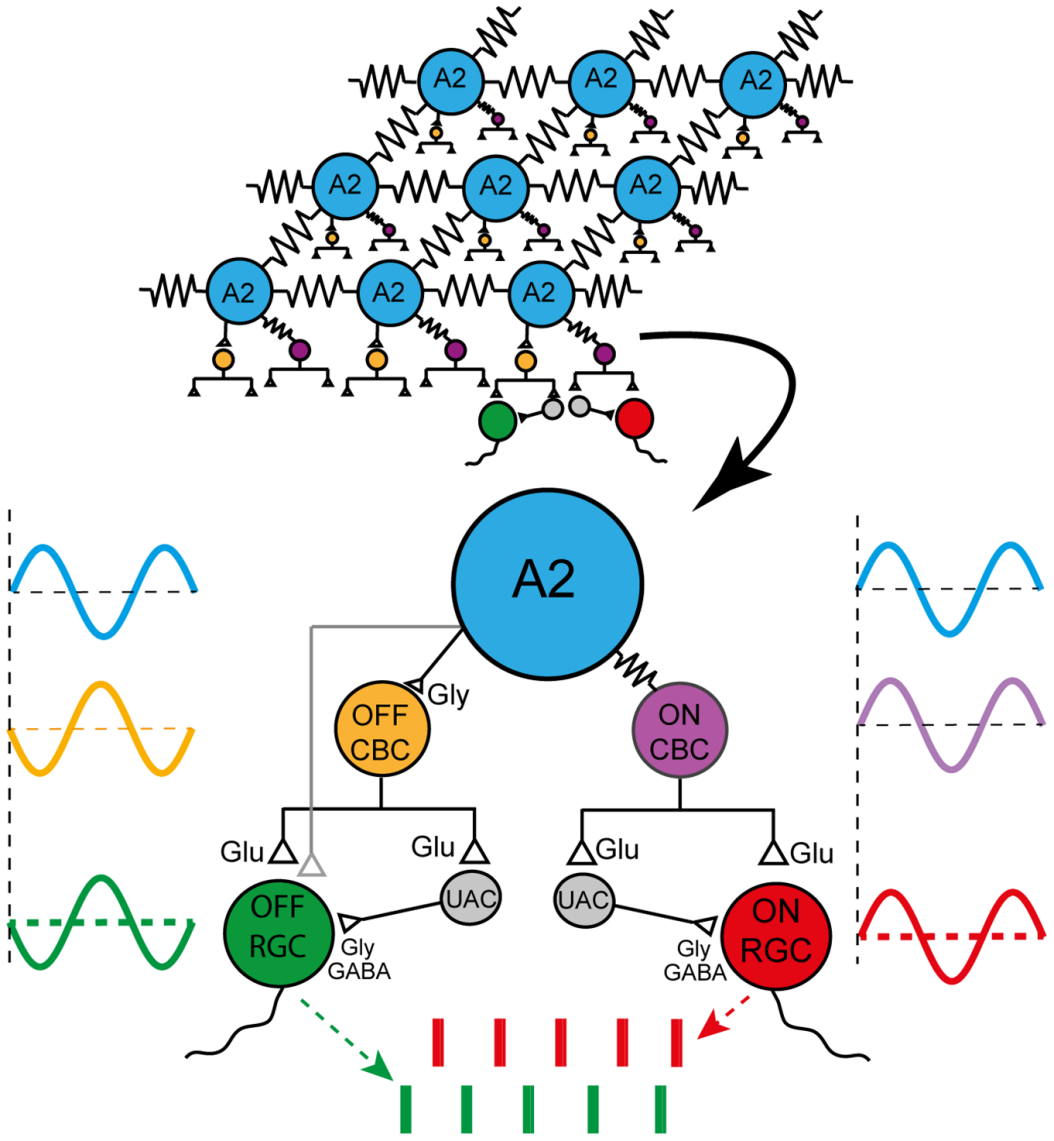
Schematic diagram of electrically and chemically mediated synaptic interactions that can account for the observed results in RD1 retina. Membrane potential oscillations (blue sinusoid) generated by a voltage-gated Na^+^ channel dependent mechanism originate in the network of electrically coupled A2 amacrine cells. The rhythmic fluctuations in membrane voltage cause oscillations in glycine release and strength of excitatory electrical transmission resulting in out of phase variations in membrane potential of OFF (orange sinusoid) and ON (purple sinusoid) cone bipolar cells (CBCs), respectively. The resulting variations in the strength of glutamatergic transmission stimulates oscillations in the membrane potential of: (1) OFF and ON RGCs thus accounting for out of phase generation of spike activity in un-like type RGCs (bottom raster trains) and (2) unidentified glycinergic or GABAergic amacrine cells (UACs) to give rise to oscillatory inhibitory synaptic input to alpha RGCs.

The specifications that these results impose are potentially satisfied by the properties of the A2 amacrine cell network that links the ON and OFF retina pathways (reviewed by [28]). The resting potential of A2s in RD1 retina slices oscillates steadily at ∼10 Hz, even when fast synaptic transmission is blocked (Fig. 7). Thus it is reasonable to propose that A2 oscillations propagate through electrical synapses to ON cone bipolars (Fig. 8) to drive rhythmic glutamatergic excitation of ON RGCs and inhibitory amacrine cells, which could account for the oscillations in inhibitory synaptic currents recorded from ON RGCs [5], [8]. Rhythmic A2 outputs also could contribute to the ∼10 Hz oscillations in excitatory synaptic current and spike discharge in OFF RGCs by causing oscillations in glycine release and synaptic inhibition of OFF cone bipolar cells and RGCs [28]–[30] (Fig. 8).

### All – On Cone Bipolar Coupled Network

The proposed circuitry represents a collaboration between A2 amacrine cells and ON cone bipolar cells (CBCs) that are joined together in an electrically coupled network of A2s electrically coupled to each other and to ON cone bipolars. Results showing that MFA, a gap junction blocker, eliminates the oscillations in synaptic inputs to RGCs [8] as well as the local oscillations in the extracellularly recorded field potentials [10], [12] attests to the importance of electrical coupling in the generation of oscillatory activity.

Since voltage oscillations are present in cone but not rod bipolar cells in RD1 retina [8] our results are consistent with the hypothesis that a pacemaker locus in the A2/ON CBC network spontaneously generates oscillations in membrane voltage that propagate by electrical coupling to ultimately entrain 10 Hz electrical activity in a population of bipolar, amacrine and ganglion cells in a patch of RD1 retina. The correlation between the spike trains recorded from pairs of cells was variable in that the time delay (phase) between synchronous bursts in the two cells fluctuated and in some cases included phase reversals and/or transient period of asynchronous spike activity (Fig. 4). It is possible that the pacemaking locus moves within the A2 network in a manner that resembles the random initiation and migration of the LFP across the RD1 retina reported previously [10]. In this scenario shifts in the location of the pacemaker would disrupt the coherence of the network's oscillations causing variations in the phase relation between synchronous activities in paired recordings as the cellular path by which the common presynaptic oscillation reaches the two cells in the recorded pair changes.

### Biophysical source of oscillations

The precise biophysical mechanism underlying the oscillations observed in the RD1 retina remains unknown, though the fact that oscillations in A2s and ON cone bipolars persist when Ca^2+^ channels are blocked indicates that the oscillations do not arise from either regenerative Ca^2+^-spikes or from network synaptic interactions ([8]; also Fig. 7). A recent study of WT A2s demonstrated that these neurons are capable of generating bursts of Na^+^-channel dependent spikes via electrically compartmentalized interactions between Na^+^ and K^+^ conductances [26]; this is consistent with the elimination of oscillatory activity in A2s in the RD1 retina by TTX (Fig. 7).

This result, however, is at odds with two previous reports that the 10 Hz oscillations in LFPs, which are seen in RD1 but not WT retina, are not blocked by TTX [10], [12]. The variations in the amplitude of the LFP are attributed to extracellular charge displacements in response to rhythmic fluctuation in the membrane potential of a population of electrically coupled presynaptic neurons. The coupled network that drives the oscillation in LFP is assumed to be the A2 network since it is the most densely populated presynaptic-electrically coupled network in the inner retina, it is close to where the field potential is recorded by extracellular macro-electrodes on the retina surface and it is generated intrinsically, i.e. persists in the present of synaptic blockade (Fig. 7). We do not have an explanation for why TTX blocked A2 oscillations in single cell recordings (Fig. 7) but not LFP oscillations in multielectrode array recording that are thought to be generated by oscillation in A2 membrane potential. It is possible that LFP is not the summed extracellular expression of voltage changes in the A2 network, but it is hard to attribute it to any other source. If TTX blocked oscillations in A2s one would expect that it would also block oscillation through out the A2 – ON CBC coupled network. It is also possible that in the concentration of TTX used in LFP recording experiments was not sufficient to block A2 oscillations. This would suggest that 0.5 µM TTX in a retina slice is more effective in blocking Na^+^ channels in the present experiments than 0.1–0.2 µM TTX in whole mount retina on a MEA. This suggestion draws some support from the fact in one of the MEA studies 0.2 µM TTX reduced but did not abolish spontaneous spike activity [10].

Whatever the ionic mechanism for generating oscillations, the natural tendency for A2s to oscillate is likely to be reinforced by them being joined together in an electrically coupled network. There are numerous examples of neurons with resting potentials that are stable in isolation but become oscillatory when connected to a coupled network. An explanation for this, which is supported by computer simulation [31], is that electrical coupling weakens the feedback interactions that keep things under control by allowing some fraction of the cell's feedback signal to leak out and be dispersed in the inter-cellularly interconnected syncytium. In this way the cell's feedback mechanisms has less tight control over the events designed to dampen or prevent oscillations.
